# Phytochemical and Biological Screening of Leaf, Bark and Fruit Extracts from *Ilex dipyrena* Wall.

**DOI:** 10.3390/life11080837

**Published:** 2021-08-16

**Authors:** Amjad Ali, Atif Ali Khan Khalil, Fazli Khuda, Nausheen Nazir, Riaz Ullah, Ahmed Bari, Adnan Haider, Syed Babar Jamal, Sareer Ahmad, Zahid Khan, Syed Muhammad Mukarram Shah, Syed Wadood Ali Shah, Abdul Nasir, Abid Ali, Muhammad Zahoor, Samin Jan

**Affiliations:** 1 Department of Botany, Islamia College University, Peshawar 25120, Khyber Pakhtunkhwa, Pakistan; amjad1990.aa48@gmail.com (A.A.); sareerkhan2020@gmail.com (S.A.); 2 Department of Botany, University of Malakand, Dir (Lower), Chakdara 18800, Khyber Pakhtunkhwa, Pakistan; 3 Department of Biological Sciences, National University of Medical Sciences, Rawalpindi 46000, Punjab, Pakistan; atif.ali@numspak.edu.pk (A.A.K.K.); adnan.haider@numspak.edu.pk (A.H.); babar.jamal@numspak.edu.pk (S.B.J.); 4 Department of Pharmacy, University of Peshawar, Peshawar 25120, Khyber Pakhtunkhwa, Pakistan; azlikhuda2012@uop.edu.pk; 5 Department of Biochemistry, University of Malakand, Dir (Lower), Chakdara 18800, Khyber Pakhtunkhwa, Pakistan; nausheen.nazir@uom.edu.pk (N.N.); mohammadzahoorus@yahoo.com (M.Z.); 6 Department of Pharmacognosy, College of Pharmacy, King Saud University, Riyadh 11451, Saudi Arabia; rullah@ksu.edu.sa; 7 Department of Pharmaceutical Chemistry, College of Pharmacy, King Saud University, Riyadh 11451, Saudi Arabia; abari@ksu.edu.sa; 8 Department of Pharmacognosy, Faculty of Pharmacy, Federal Urdu University of Arts Science and Technology, Karachi 75300, Sindh, Pakistan; Zahidkhan@fuuast.edu.pk; 9 Department of Pharmacy, University of Malakand, Dir (Lower), Chakdara 18800, Khyber Pakhtunkhwa, Pakistan; mukaramshah@uoswabi.edu.pk (S.M.M.S.); pharmacistsyed@gmail.com (S.W.A.S.); 10 Department of Molecular Science and Technology, Ajou University, Suwan 16499, Korea; anasirqau@gmail.com; 11 Khyber Teaching Hospital, Peshawar 25120, Khyber Pakhtunkhwa, Pakistan; abidjan81@gmail.com

**Keywords:** *Ilex dipyrena* wall., antioxidant, analgesic, phytochemicals, GC-MS analysis

## Abstract

The Aquifoliaceae is an important family and has been used traditionally for some time. One of the members of this family is the *Ilex dipyrena* wall, which itself possesses a potential medicinal importance. This plant is traditionally used for the treatment of various ailments including pain, swelling, burns, and fever. The current study was designed to screen out the antioxidant and analgesic potential of this plant and to verify its traditional uses, along with its phytochemical profile. Extracts were subjected to antioxidant, analgesic, and phytochemical analysis using DPPH, chemical-induced (acetic acid and formalin) nociception models and GC-MS analysis, respectively. The leaf, bark, and fruit extracts showed significant antioxidant activity compared to that of standard. Likewise, all the extracts demonstrated significant (*p* < 0.01) analgesic activity in a mice model. In acetic acid induced analgesia, the leaf, bark, and fruit extracts caused 51.64, 56.13 and 59.52% inhibition, respectively at a dose of 100 mg/kg while at 200 mg/kg it showed 83.01, 71.69 and 75.47% inhibition, respectively. In Formalin-induced paw-licking assay, fruit extract showed 59.42 and 64.19% inhibition at 200 mg/kg dose in the first and second phase, respectively. The GC-MS analysis revealed the presence of cathinone, phenylpropanolamine, dl-phenylephrine, amphetamine, myristic acid, and palmitic acid. Results of the study suggest that crude extracts from different parts of this plant may be a useful source for the development of novel analgesics. However, further investigation in terms of isolation of bioactive compounds and their toxicological evaluations are needed to validate the observed results.

## 1. Introduction

From immemorial times, natural products, (including animals, plants, and minerals) have been the basis of treating ailments. Nature has been a great source of therapeutic agents since the dawn of human civilization and has continued to provide novel therapies to mankind [1]. With the observational and scientific efforts of scientists, the current allopathy, or modern medicine, came into practice. However, the mainstay of its advancement remains rooted in traditional therapies and medicines. Medicine’s history has a lot of strange therapies. However, ancient human ancestral wisdom has been and will continue to be an important source of therapeutics and future medicines [2]. The prospect of natural products and drug breakthroughs will be more personalized, holistic, and make use of modern and ancient therapy tools in a harmonizing approach so that utmost benefits can be delivered to the community and particuar patients [3].

The genus *Ilex* is a member of Aquifoliaceae family, having 600 species, found mostly in tropical temperate regions. Generally, they are evergreen deciduous trees and sometimes shrubs. Most of the species are used extensively for the various disease therapies in traditional herbal medicine worldwide due to the presence of saponins [4]. Flavonoids [5], aldehydes [6], triterpenes, hemiterpene glycosides, anthocyanins, alkanes, hexyl esters, pentyl esters, and other lipophilic chemical compounds were previously identified in different species of the genus. In-vitro and in-vivo studies have previously shown that *Ilex latifolia* extracts have potent anti-inflammatory and antinociceptive properties [7]. A purified saponin fraction derived from the root of *I. pubescens* demonstrated a significant analgesic effect in both visceral and central nociceptive models [8]. In addition, in South America, the leaves of *I. paraguariensis* (mate tree) are widely used for making an infusion tea known as mate, which contains a large amount of caffeine and theobromine. *I. tarapotina* and *I. vomitoria* are used for making stimulatory beverages. Other species of *Ilex* (*I. cornuta, I. aquifolium, I. crenata* and *I. opaca*), commonly called “*hollies*”, are usually cultivated for Christmas tree production or landscaping [9,10].

*Ilex dipyrena* Wall. of the family Aquifoliaceae is an evergreen tree that grows to about 10 m height and is widely distributed in tropical regions of the world, including India and Pakistan [11]. This plant contains several phytochemicals with cembratriene and solanesol as the major constituents. Traditionally, it has been used for the treatment of many aliments such as cancer, inflammation, pain, cardiac ailments, and several infectious disorders. According to reported data, this plant has only been tested for its antimicrobial activities [11]. To the best of our knowledge, no other pharmacological activity has been reported so far. Based on its traditional uses, we explored the phytochemical and toxicity profile of the study plant along with antioxidant and analgesic activities. The results of the study will provide a scientific base for the folkloric reported medicinal characteristics of the plant.

## 2. Materials and Methods

### 2.1. Chemicals

Alliance Pharmaceuticals and Aries Pharmaceuticals, Peshawar, KPK, Pakistan, donated diclofenac sodium and tramadol. 2,2-Diphenyle-1 Picryl-hydrazyle (DPPH), hydrochloric acid (HCl), Sulphuric Acid (H_2_SO_4_) sodium hydroxide (NaOH), magnesium ribbon, petroleum ether, chloroform, *n*-pentane were purchased from Sigma-Aldrich, Germany. Merck (Darmstadt, Germany) provided methanol, formalin, and acetic acid.

### 2.2. Plant Material Collection

Whole plants, including leaves, bark, and fruits were collected in May 2015 from District Shangla, Khyber Pakhtunkhwa, Pakistan. Plants were identified by local people by their local names and were then authenticated by plant taxonomist Dr. Jahandar Shah. The plants were washed with tap water and then dried in shade. After drying, the materials were subjected to pulverization through a mechanical grinder.

### 2.3. Extraction

Maceration of powder plant leaves with methanol (480 g, 1.5 L), bark (700 g, 2 L) and fruits (360 g, 1.5 L) was separately carried out for 15 days. The methanolic extract was then filtered and concentrated by the process of evaporation using rotary evaporator (Rotary Vacuum Evaporator Laborota-4010, Heidolph Co., Schwabach, Germany) under reduced pressure at 40 °C. After complete evaporation of the solvent, greenish (1700 mg), black (213 mg) and light yellow (177 mg) crude methanolic extracts were obtained for leaves, bark, and fruits, respectively. The crude methanolic extracts were stored in glass vials at 4 °C for further use.

### 2.4. Phytochemical Screening

The crude extracts of leaves, bark, and fruits were subjected to qualitative chemical tests for the identification of phytochemicals such as tannins (ferric chloride test), saponins (froth and emulsion test), flavonoids (sodium hydroxide and magnesium ribbon test), terpenoids, and sterols (chloroform and sulphuric acid test) [12].

### 2.5. Gas Chromatography-Mass Spectrometry (GC-MS) Analysis

GC-MS analysis of samples (bark, leaf, and root extract) were carried out using an Agilent USB-393752 gas chromatograph (Agilent Technologies, Palo Alto, CA, USA) equipped with an HHP-5MS 5% phenylmethylsiloxane capillary column (30 m × 0.25 mm × 0.25 μm film thickness; Restek, Bellefonte, Pennsylvania, USA) outfitted with an Agilent HP-5973 mass selective detector (Agilent Technologies, Palo Alto, CA, USA) in the electron impact mode (Ionization energy: 70 eV). Initially the oven temperature was set at 70 °C for 1 min, and then the temperature was increased to 180 °C at the rate of 6 °C/min for 5 min. The final temperature was 280 °C that was achieved in 20 min at the rate of 5 °C/min, while the injector temperature was 220 °C and detector temperatures were 290 °C. Samples after required dilutions (1/1000 in *n*-pentane, *v*/*v*) in 1 μL volume were injected manually in the split-less mode. Helium was used as carrier gas at a flow rate of 1 mL/min which propelled the compounds. The eluted fractions from GC column were chemically ionized before their entries into mass spectrometer. The ions separated on the basis mass-to-charge (*m/z*) ratios were then identified.

### 2.6. Identification of Components

Compounds were identified by comparison of their retention times with those of authentic compounds reported in the literature. Further identification was done through the spectral data obtained from the Wiley and NIST libraries while comparisons of the fragmentation pattern observed in the mass spectra with data published in the literature was also used as an identification tool [13,14].

### 2.7. Antioxidant Activity

DPPH solution was prepared by dissolving (24 mg) in 100 mL of methanol. Methanolic stock solution of plant extract with the concentration of 1 mg/mL with further dilution to the concentrations of 500, 250, 125, 62.5 μg/mL. From each sample, 0.1 mL diluted solution was mixed with 3 mL of DPPH in methanol. Incubation of the solution was carried out at 23 °C for 30 min. After incubation absorbance was taken at 517 nm. Ascorbic acid was taken as a positive control [15]. With the help of the following equation, percent free radical scavenging activity was calculated a% free radical scavenging activity = A (Control absorbance) − B (sample absorbance)/A (control absorbance) × 100.

### 2.8. Animals and Ethical Approval

Male Balb/C mice weighing 20–25 g was obtained from the National Institute of Health in Islamabad, Pakistan. The animals were isolated in an animal house under standard laboratory conditions (25 °C, 55–65 percent relative humidity, and a 12 h light/12 h dark cycle), with a standard feed and ad libitum water. In addition, after the experiments, the animals were sacrificed using isoflurane euthanasia. All protocols employed in the study were authorized by the University’s Departmental Ethical Committee (Pharm/EC-Id/37-12/14) in accordance with the University of Malakand’s Animal Byelaws 2008, Scientific Procedures Issue-I.

### 2.9. Acute Toxicity Test

Mice were taken as test animals that were divided into control and test groups (6 mice in each group). Administration of the plant samples was carried out with various doses in the range of 250 to 2000 mg/kg per kg body weight of mice to the respective test groups. After receiving the doses, the mice were observed for 72 h, followed by observation for 14 days with free access to food and water. For two weeks, the animals were watched daily for signs of convulsions, tremor, diarrhoea, salivation, lethargy, and sleeping. As part of the weekly observation, the body weight was also measured [16].

### 2.10. Analgesic Activity

#### 2.10.1. Acetic Acid-Induced Writhing Test

The mice in the experimental groups (*n* = 8) received crude extracts (i.p) of leaf, bark, and fruits at various dose concentrations (100 and 200 mg/kg b.w for leaf, bark, and fruit) 30 min prior to acetic acid administration (0.6%, 10 mL/kg, i.p). The negative control group received 10 mL/kg of 1% solution of Tween 80 (1%, *v*/*v*) and the positive control group received 10 mg/kg (i.p) of diclofenac sodium. The intensity of nociception was recorded in the number of writhes produced within 30 min of acetic acid administration [17].

#### 2.10.2. Formalin Test

The experimental mice groups (*n* = 8) received crude extract (i.p) of leaf, bark, and fruits at different dose concentrations 1 h prior the treatment of animals in the respective groups were treated with formalin (1%, 50 μL) on the right hind paw. The treated paw of mice was observed through a plexiglass box for 30 min and the paw licking of mice was recorded in seconds in two phases, 0–5 min (neurogenic pain), and 15–30 min (inflammatory pain) [18].

### 2.11. Statistical Analysis

The data was expressed as mean ± SEM. Graph Pad Prism 5 version 5.01 was used for statistical analysis using one-way ANOVA followed by Dunnett’s test. The results were considered to be significant at *p* < 0.05.

## 3. Results and Discussion

### 3.1. Phytochemical Screening

Phytochemical screening is an important tool for identifying secondary metabolites of a medicinal plant, as most of them are responsible for important therapeutic actions [19]. It has been reported that saponins are among the most important compounds responsible for a variety of biological activities [20]. Saponins are reported for their antimicobial, antioxidant, cytotoxic, phytotoxic, antitumer, antispasmodic, antidiabetic, and anthelmintic activities [21]. Besides this, crude saponins have been used for their anthelmintic, anticancer, and insecticidal activities [22].

Phytochemical screening of *I. dipyrena* leaves, bark, and fruits crude methanolic extracts revealed that they are highly rich in phyto-constituents (Table 1). Crude extract obtained from the leaves and bark contain high number of phenols, tannins, saponins, flavonoids, steroids, and terpenoids; however, the fruit extracts do not contain saponins. Results of phytochemical analysis of the crude methanolic extracts obtained from leaves, bark, and fruits of the *I. dipyrena* confirm that the plant is rich in secondary metabolites. In addition, our results on phytochemical screening were also consistent with the results of other species of *Ilex* genus [23].

### 3.2. GC-MS Analysis

**Figure 1:**
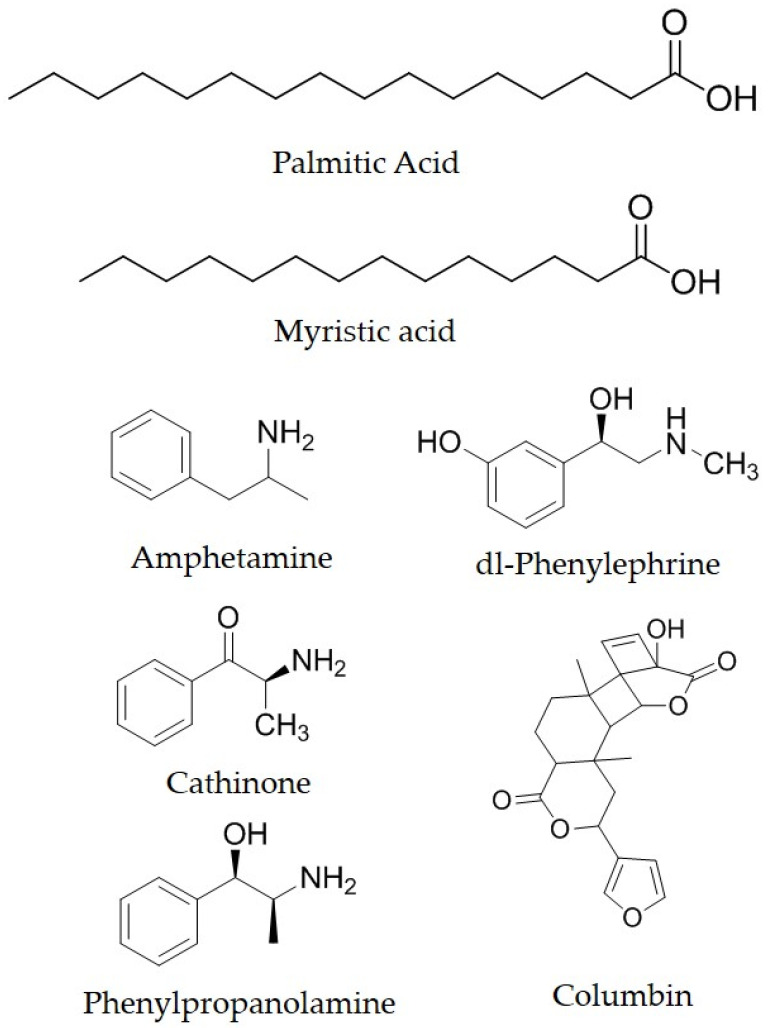
Structures of some important components of *I. dipyrena* from GC-MS analysis.

The GC-MS analysis of three different samples of *I. dipyrena* was performed for the identification of major phytochemical components. The list of all the identified compounds in the bark, leaf, and fruit extract has been compiled in Table 2, Table 3 and Table 4, respectively. A total number of eighteen compounds have been identified in bark, twenty-three in leaf, and thirty-three in the fruit of *I. dipyrena*. Among these cathinone, amphetamine, myristic acid, and palmitic acid have been detected in all the test samples (Figure 1).

### 3.3. Antioxidant Activity

**Figure 2:**
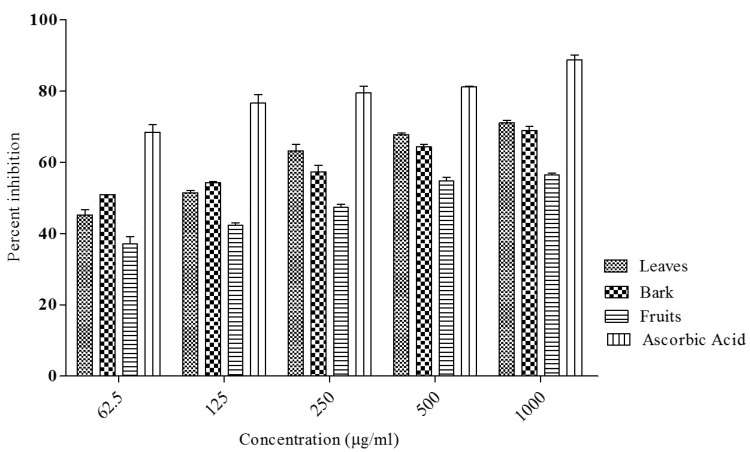
Percent DPPH inhibition activity of crude methanolic extract of *I. dipyrena* leaves, bark and fruits.

In present study, crude extracts from the *I. dipyrena* leaves, bark, and fruits were investigated for their antioxidant potentials using DPPH free radical inhibition assay. Percent (%) free radical scavenging activity and IC_50_ values of all the tested samples are summarized in Figure 2 and Table 5, respectively. The test samples showed dose-dependent activity. Leaf extract caused 45.23 ± 1.53, 51.52 ± 0.54, 63.27 ± 1.83, 67.82 ± 0.43 and 71.11 ± 0.73% inhibition at 62.5, 125, 250, 500, and 1000 µg/mL concentrations, respectively. The IC_50_ value for leaf extract was 113 µg/mL. Similarly, the fruit extract showed 37.18 ± 1.92, 42.43 ± 0.54, 47.42 ± 0.82, 54.79 ± 1.13 and 56.45 ± 0.53% free radical scavenging activity at the same concentrations and IC_50_ value was found to be 327 µg/mL. *I. dipyrena* bark extract exhibited almost same antioxidant activity i.e., 51.00 ± 0.00, 54.32 ± 0.34, 57.38 ± 1.74, 64.35 ± 0.75 and 69.00 ± 1.16% DPPH inhibition at 62.5, 125, 250, 500 and 1000 µg/mL concentrations, respectively with IC_50_ of 41 µg/mL. Ascorbic acid showed 68.48 ± 2.21, 76.64 ± 2.43, 79.53 ± 1.86, 81.22 ± 0.16, and 88.83 ± 1.38% inhibition at 62.5, 125, 250, 500, and 1000 µg/mL concentrations, respectively with IC_50_ value < 0.1 µg/mL. The moderate antioxidant activity of the crude extract of *I. dipyrena* leaves, bark, and fruits can be linked to the presence of phytochemicals, especially phenols.

Natural antioxidants and their health advantages have received a lot of attention in recent years. Antioxidant-based drug formulations are used to prevent and treat a wide range of complex diseases. Plants are a significant source of natural antioxidants; they produce a diverse range of secondary metabolites with antioxidative activities and therapeutic potential [24,25]. Polyphenols are the plant’s most abundant antioxidant compounds. Their antioxidant activity is based on their redox properties, which allow them to act as reducing agents, hydrogen donors, and singlet oxygen quenchers. The presence of reductants, which act as antioxidants by breaking the free radical chain by donating a hydrogen atom or preventing peroxide formation, is generally associated with the presence of reducing ability. Flavonoids, phenolic acids, stilbenes, tannins, coumarins, lignans, and lignins are common phenolic compounds found in medicinal plant tissues. These substances have a variety of biological effects [24,25].

Plants continue to be the primary source of natural antioxidants. Several plants have been identified as having antioxidant activity. *Hyssopus officinalis, Angelica pancicii, Angelica sylvestris, Laserpitium latifolium, Achillea grandifolia, Achillea crithmifolia, Artemisia absinthium*, and *Tanacetum parthenium* are among the most prominent. Based on our results, the study plant may be used as a food supplement for the treatment of several disorders.

### 3.4. Acute Toxicity

Acute toxicity tests in a proper animal model is of vital importance for medicinal plants before investigating them for in-vivo pharmacological studies. This study identifies the doses above which toxic effects and lethality occurs. Thus, acute toxicity tests in animals validated the safe dose for further animal studies. Acute toxicity activity of crude methanolic extracts of *I. dipyrena* leaves, bark, and fruits was investigated in mice in two stages and the results are tabulated in Table 6. In the first stage, animals were given test samples i.p at 10,100 and 1000 mg/kg body weight and were kept under observation for toxic effects or lethality. In the first stage, no test sample i.e leaves, bark, and fruits, was found lethal or toxic up to 1000 mg/kg body weight (i.p). In the second stage acute toxicity study, animals were given test samples at 1250, 1500 and 1750 mg/kg body weight. The bark and fruits crude methanolic extracts were found safe at all three doses and results confirmed that both are safe at up to 1750 mg/kg body weight i.p doses.

While the leaves crude methanolic extract sample was found to be different from that of the bark and fruits samples in the second stage, at 1250 mg/kg body weight i.p dose, it caused 50% of the mice death. Similarly, at 1500 mg/kg body weight i.p dose, it caused the death of all experimental animals. The results show that bark and fruit crude extract samples are safe and nontoxic up to 1750 mg/kg body weight i.p dose. While leaves crude methanolic extract sample are safe up to 1000 mg/kg body weight i.p dose but caused 50% animal deaths at 1250 mg/kg body weight i.p dose, at 1750 mg/kg body weight i.p dose it caused the death of all animals.

### 3.5. Analgesic Activity

Analgesic activity of *I. dipyrena* fruits, leaves, and bark crude extract was investigated using two different models. In acetic acid induced analgesic activity, leaves extract caused 51.64 and 83.01% inhibition at a dose of 100 and 200 mg/kg, respectively. Bark extract caused 56.13 and 71.69%, while fruits extract caused 59.52 and 75.47% inhibition at 100 and 200 mg/kg body weight, respectively. Diclofenac sodium showed 80.83% analgesia inhibition at a dose of 10 mg/kg (Table 7).

In Formalin-induced paw-licking test, leaves extract demonstrated 17.60 and 49.65% activity at a dose of 100 mg/kg. Similarly, at 200 mg/kg the extract showed 36.71 and 59.40% at both phases, respectively. Bark extract demonstrated comparatively low analgesic potential and could only cause 0.039 and 17.43% protection at 100 mg/kg dose with 0.059 and 23.93% protection at first and second phase, respectively. Fruit extract showed 42.11 and 55.90% inhibition at 100 mg/kg dose while at 200 mg/kg it exhibited 59.42 and 64.19% protection. Indomthacin was used as standard and showed 60.36 and 78.25% inhibition at a dose of 10 mg/kg (Table 8).

Leaves and fruits crude extracts showed profound analgesic activity in acetic acid induced abdominal writhing test while moderate antinociceptive potential in formalin induced paw liking test in mice. The analgesic activity observed for the various samples of *I. dipyrena* could be linked with the presence of variety of compounds in the extract. The GC-MS analysis of the crude extract showed numerous compounds. The thorough literature review of the GC-MS data revealed the presence of some very important analgesic compounds viz; cathinone, Phenylpropanolamine, dl-phenylephrine, amphetamine, myristic acid, and palmitic acid columbin. The cathinone has been reported for its prolonged analgesic activity [26]. Similarly, phenylpropanolamine belongs to the category of adrenoreceptor agonist and this compound has been evidenced with potentiation of opioid antinociception via the adrenoreceptors [27]. Among these compounds the dl-phenylephrine actually prolong the spinal anesthesis caused by the lidocaine, which may also be correlated with the analgesia [28]. Moreover, columbin is of special interest due to its structural similarity to the known kappa-opioid receptor agonist salvinorin A [29]. One of the previous reports shows that the D-amphetamine possesses analgesic activity which is somewhat comparable with ibuprofen [30]. In the same way the palmitic acid provides a scaffold for the analgesic compounds [31]. Moreover, GC-MS analysis of the extracts confirmed the presence of several chemical constituents in the bark, leaf, and fruit of *I. dipyrena* that may be involved in analgesic activity. Some of the most common components of essential oils such as myristic acid, palmitic acid, capric acid, stearic acid, arachic acid, and cathinone have been identified in the essential oil of *I. dipyrena* (Figure 1). Some of these chemical constituents have been reported by other researchers to possess analgesic and psychoactive activities. Results of the study suggest that the extract possesses potent analgesic activity and therefore may be considered in the development of potentially safe and effective therapy.

## 4. Conclusions

In this study, we investigated antioxidant and analgesic activities of *I. dipyrena* leaf, bark, and fruit extracts. The analysis of obtained results suggested that *I. dipyrena* leaf, bark, and fruit extracts have considerable antioxidant and analgesic properties. Furthermore, *I. dipyrena* phytochemical screening and GC-MS analysis revealed the existence of medicinally significant secondary metabolites. Moreover, the chemically unknown chemicals found in *I. dipyrena* could be a source of novel drugs, necessitating a full chemical analysis to separate bio-active ingredients and follow their biological activity. From our investigations of obtained results, we concluded that the genus Ilex can play a pivotal role in modern medicine in the near future.

## Figures and Tables

**Table 1:** Results of phytochemical screening of crude methanolic extracts of leaves, bark, and fruits of *I. dipyrena*.

Phytochemicals	Leaves	Bark	Fruit
Tannins	++	++	++
Saponins	+++	+++	−
Flavonoids	+++	+++	+++
Phenolics	+++	+++	+++
Steroids	+++	+++	+++
Terpenoids	+++	+++	+++

Notes: +++: Strong positive test; ++: Weak positive test; −: Absent.

**Table 2:** List of compounds in the bark of *I. dipyrena*.

S.No	Compound Label	Common Name	CAS	Mol. wt	Formula
1	1-propanone, 2-amino-1-penyl-, (S)-	Cathinone	71031-15-7	149	C_9_H_11_NO
2	Propanamide -3-(3,4-dimethylphenslsulfonyl)-	NF	0-0-0	241	C_11_H_15_N0_3_S
3	Benzeneethanamine, alphamethyl-	Amphetamine	60-15-1	135	C_9_H_13_N
4	Phenylpropanolamine	NF	492-41-1	151	C_9_H_13_NO
5	Benzeneethanamine, 3,4-benzyloxy-2-5-diflouro-beta-hydroxy-N-methyl-	NF	0-0-0	399	C_23_H_23_F_2_NO_3_
6	Methylenedioxy-amphetamine	Tenamfetamine	4764-17-4	179	C_10_H_13_N0_2_
7	Phenol, 4-(2aminopropyl)-(+/−)-	Paradrine	1518-86-1	151	C_9_H_13_NO
8	Tetraacetyl-d-xylonic nitrile	NF	0-0-0	343	C_14_H_17_NO_9_
9	Butanal, 3-methyl-	Isovaleraldehyde	590-86-3	86	C_5_H_10_O
10	Butanal, 3-hydroxy-	Aldol	107-89-	88	C_4_H_8_0_2_
11	1,3,4-trihydroxy-5-oxo-cyclohexanecarboxylic acid	NF	0-0-0	190	C_7_H_10_O_6_
12	Propanenitrile, 3-amino-2,3-di (hydroxymino)	NF	0-0-0	128	C_3_H_4_N_4_0_2_
13	Pentadecanoic acid, 14-methyl-, methyl ester	NF	5129-60-2	270	C_17_H_3_40_2_
14	Methyl tetradecanoate	Myristic acid	124-10-7	242	C_15_H_30_O_2_
15	Tridecanoic acid, methyl ester	Methyl tridecanoate	1731-88-0	228	C_14_H_28_O_2_
16	Hexadecanoic acid, 15-methyl-, methyl ester	NF	6929-04-0	284	C_18_H_36_O_2_
17	Decanoic acid	Capric acid	334-48-5	172	C_10_H_20_O_2_
18	n-Hexadecanoic acid	Palmitic acid	57-10-3	256	C_16_H_32_O_2_

NF: Not found.

**Table 3:** List of compounds in the leaf of *I. dipyrena*.

S.No	Compound Label	Common Name	CAS	Mol. wt	Formula
1	*p*-Xylene	*p*-Xylene	106-42-3	106	C_8_H_10_
2	*o*-Xylene	*o*-Xylene	95-47-6	106	C_8_H_10_
3	Benezeneethanol, Alpha, beta-dimethyl-	3-phenyl-2-butanol	52089-32-4	150	C_10_H_4_O
4	Ethylbenezene	Ethylbenezene	100-41-4	106	C_8_H_10_
5	1,3-Cyclopentadiene, 5-(1-methylethylidene)-	NF	2175-91-9	106	C_8_H_10_
6	N-(3,5-Dinitropyridin-2-yl)-I. Aspartic acid	NF	35899-60-6	399	C_23_H_23_F_2_NO_3_
7	Benzeneethanamine, alphamethyl-	Amphetamine	60-15-1	135	C_9_H_13_N
8	1-propanone, 2-amino-1-penyl-, (S)-	Cathinone	71031-15-7	149	C_9_H_11_NO
9	2H-pyran-3-ol, 6-ethenyltetrahydro-2,2,6-trimethyl-	NF	14049-11-7	170	C_10_H_18_O_2_
10	7-Octen-2-ol, 2-methyl-6-methylene-	Myrcenol	543-39-5	154	C_10_H_18_0
11	Cyclopropanemethanol, 2-isopropylidene-alpha.-methyl-	NF	17219-1-1	126	C_8_H_14_O
12	2-Furanmethanol, 5-ethenyltetrahydro-alpha,alpha,5-trimethyl-, cis-	NF	5989-33-3	170	C_10_H_18_O_2_
13	Hexadecanoic acid, methyl ester	NF	112-39-0	270	C_17_H_34_O_2_
14	Hexadecanoic acid, 15-methyl-, methyl ester	NF	6929-04-0	284	C_18_H_36_O_2_
15	Tetradecanoic acid	Myristic acid	544-63-8	228	C_14_H_28_O_2_
16	Hexadecanoic acid	Palmitic Acid	57-10-3	256	C_16_H_32_O_2_
17	Octadecanoic acid	Stearic acid	57-11-4	284	C_18_H_36_O_2_
18	Pentadecanoic acid	NF	1002-84-2	242	C_15_H_30_O_2_
19	9,12,15-Octadecatrienoic acid, methyl ester, (Z,Z,Z)-	NF	301-0-8	292	C_19_H_32_O_2_
20	11,14,17-Eicosatrienoic acid, methyl ester	NF	55682-88-7	320	C_21_H_36_O_2_
21	9,12,15-Octadecatrien-l-ol, (Z,Z,Z)-	NF	506-44-5	264	C_18_H_32_O
22	Methyl (Z)-5,11,14,17-eicosatetraenoate	NF	59149-1-8	318	C_21_H_34_O_2_
23	Cis,cis,cis-7,10,13-Hexadecatrienal	NF	56797-43-4	234	C_16_H_26_O

NF: Not found.

**Table 4:** List of compounds in the fruit of *I. dipyrena*.

S.No	Compound Label	Common Name	CAS	Mol. wt	Formula
1	*p*-Xylene	*p*-Xylene	95-47-6	106	C_8_H_10_
2	*o*-Xylene	*o*-Xylene	95-47-6	106	C_8_H_10_
3	Benezeneethanol, Alpha, beta-dimethyl-	3-phenyl-2-butanol	52089-32-4	150	C_10_H_14_O
4	1,3-Cyclopentadiene, 5-(1-methylethylidene)-	NF	2175-91-9	106	C_8_H_10_
5	Ethylbenzene	Ethylbenzene	100-41-4	106	C_8_H_10_
6	Phenylpropanolamine	NF	492-41-1	151	C_9_H_13_NO
7	Benzeneethanamine, alpha,-methyl-	Amphetamine	60-15-1	135	C_9_H_l3_N
8	1-propanone, 2-amino-1-penyl-, (S)-	Cathinone	71031-15-7	149	C_9_H_11_NO
9	Benzenemethanol. 3-hydroxy-alpha-(methylamino)methyl-(+/-)-	NF	1477-63-0	167	C_9_H_l3_NO_2_
10	Metanephrine	NF	5001-33-2	197	C_l0_H_l5_NO_3_
11	Benzenemethanol,3-hydroxy-alpha-(methylamino)methyl)-, (R)-	NF	59-42-7	167	C_9_H_l3_NO_2_
12	phenylenphrine	NF	1477-63-0	167	C_9_H_l3_NO_2_
13	Pterin-6-carboxylic acid	NF	948-60-7	207	C7H_5_N_5_O_3_
14	Imidazole, 2-amino-5-[(2-carboxy)vinyl]-	NF	0-0-0	153	C_6_H_7_N_3_O_2_
15	3-Azabicyclo[3.2.2]nonane	NF	283-24-9	125	C_8_H_l5_N
16	lH-4-Azacycloprop[cd]indene, octahydro-4-methyl-	NF	16967-50-3	137	C_9_H_15_N
17	1,4.Etheno-3H.7H-benzo[,.2-c:3,4-c]dipyran-3.7-dione.9-(3-furanyl)decahydro-1-hydroxy-1a.10a-dime	Columbin	546-97-4	358	C_20_H_22_O_6_
18	3-Buten-2-one, 4-(6,6-dimethyl-1-cyclohexen-l-yl)-	NF	65133-79-1	178	C_12_H_18_O
19	trans-Dihydrophymaspermone.	NF	36203-84-6	232	C_l5_H_20_O_2_
20	Biscyclo[2.2.1]heptan-2-one, 4,7,7-trimethyl-semicarbazone	NF	24230-79-3	209	C_11_H_19_N_3_O
21	Hexadecanoic acid, methyl ester	NF	112-39-0	270	C_17_H_34_O_2_
22	Octadecanoic acid, 17-methyl-, methyl ester	NF	55124-97-5	312	C_20_H_40_O_2_
23	Pentadecanoic acid, 14-methyl-, methyl ester	NF	5129-6-2	270	C_17_H_34_O_2_
24	Tetradecanoic acid	Myristic acid	544-63-8	228	C_14_H_28_O_2_
25	n-Hexadecanoic acid	Palmitic Acid	57-10-3	256	C_16_H_32_O_2_
26	Octadecanoic acid	Stearic acid	57-11-4	284	C_18_H_36_O_2_
27	Pentadecanoic acid	NF	1002-84-2	242	C_15_H_30_O_2_
28	Eicosanoic acid	Arachic Acid	506-30-9	312	C_20_H_40_O_2_
29	13-Oxabicyclo[10.1.0]tridecane	NF	286-99-7	182	C_12_H_22_O
30	Oleyl Alcohol	NF	143-28-2	268	C_18_H_36_O
31	E-7-Tetradecenol	NF	0-0-0	212	C_14_H_28_O
32	13-Tetradecenal	NF	85896-31-7	210	C_14_H_26_O
33	9,12-Octadecadienoic acid (Z,Z)-	NF	60-33-3	280	C_18_H_32_O_2_

NF: Not found.

**Table 5:** IC_50_ values of crude methanolic extracts of leaves, fruits and bark of *I*. *dipyrena* against DPPH free radical.

Samples	IC_50_ (µg/mL)
Leaf	113
Bark	41
Fruit	327
Ascorbic acid	<0.1

**Table 6:** Acute toxicity study of fruits, bark and leaves methanolic crude extracts *I. dipyrena* in mice.

Dose (mg/kg Body Weight)			
1st stage	Group 1 (10 mg)	Group 2 (100 mg)	Group 3 (1000 mg)
Fruit	Alive	Alive	Alive
Bark	Alive	Alive	Alive
Leaves	Alive	Alive	Alive
2nd stage	Group 1 (1250 mg)	Group 2 (1500 mg)	Group 3 (1750 mg)
Fruit	Alive	Alive	Alive
Bark	Alive	Alive	Alive
Leaves	50% died	All died	-

**Table 7:** Acetic acid induced analgesic activity of the *I. dipyrena.*

Treatment/Dose	Number of Writhing	% Inhibition
Control (2% Tween 80)	53 ± 1.02	---
Leaves 100 mg	25.63 ± 1.25 **	51.64
Leaves 200 mg	09 ± 1.05 **	83.01
Bark 100 mg	23.25 ± 1.20 **	56.13
Bark 200 mg	15 ± 1.35 ***	71.69
Fruit 100 mg	21.45 ± 1.15 **	59.52
Fruit 200 mg	13 ± 1.20 ***	75.47
Diclofenac sodium (10 mg)	10.16 ± 0.70 ***	80.83

All the values were expressed as mean ± SEM (*n* = 8). ** *p* < 0.01, *** *p* < 0.001 when compared to control group (one way ANOVA followed by Dunnetts: compare all vs. control test).

**Table 8:** Formalin-induced paw-licking, analgesic activity of *I. dipyrena*.

Treatment/Dose	Licking Time (Sec)		Inhibition (%)	
	1st Phase	2nd Phase	1st Phase	2nd Phase
Control (2% Tween 80)	50.03 ± 1.63	72.00 ± 1.30	----	----
Leaves 100 mg	41.22 ± 1.12 **	36.25 ± 1.619 **	17.60	49.65
Leaves 200 mg	31.66 ± 1.125 **	29.23 ± 1.668 ***	36.71	59.40
Bark 100 mg	50.01 ± 1.65 **	59.45 ± 1.425 **	0.039	17.43
Bark 200 mg	50 ± 1.411 ***	54.77 ± 1.039 ***	0.059	23.93
Fruit 100 mg	28.96 ± 1.55 **	31.75 ± 1.441 ***	42.11	55.90
Fruit 200 mg	20.3 ± 1.05 **	25.78 ± 0.95 **	59.42	64.19
Indomethacin (10 mg)	19.83 ± 1.55 **	15.66 ± 1.542 ***	60.36	78.25

All the values were expressed as mean ± SEM (*n* = 8). ** *p* < 0.01 and *** *p* < 0.001 when compared to control group (one-way ANOVA followed by Dunnetts: compare all vs. control test).
